# Older adults’ experiences of using a wearable activity tracker with health professional feedback over a 12-month randomised controlled trial

**DOI:** 10.1177/2055207620921678

**Published:** 2020-04-26

**Authors:** Katie-Jane Brickwood, Andrew D Williams, Greig Watson, Jane O’Brien

**Affiliations:** School of Health Science, University of Tasmania, Australia

**Keywords:** Physical activity, wearables, telemedicine, older adults

## Abstract

**Objective:**

Wearable activity trackers can help older adults remain physically active. However, knowledge of the user experience during long-term use is scarce. Therefore, this study examined older adults’ experiences with, and perceptions of, wearable activity trackers combined with health professional feedback after a year’s use as part of a randomised controlled trial.

**Methods:**

Twenty older adults (73.6 ± 5.5 years) who had used a Jawbone UP24 activity tracker for 12 months during a randomised controlled trial were recruited for this study. All participants had at least one chronic condition. Acceptability data relating to activity tracker wear time was combined with focus group data to explore participants experiences of long-term activity tracker use. Data was analysed using thematic analysis.

**Results:**

The activity tracker was well-accepted with the device worn on an average of 86% of possible days and participants reported an overall positive experience. Four themes were identified: (a) increased sense of awareness of activity levels is related to motivation; (b) the level of engagement with the activity tracker influences the user experience; (c) the role of feedback from a health professional in providing ongoing support; d) the role of habits in supporting long-term behaviour change.

**Conclusions:**

The use of an activity tracker combined with health professional support can assist older adults to maintain their activity levels over 12 months. Consideration should be given to the previous technology experience of users and the design and accuracy of an activity tracker when recommending their use in a research or clinical setting.

## Introduction

Regular participation in physical activity plays an important role in maintaining health and functional independence into old age.^1–3^ Paradoxically, however, physical activity participation tends to decrease with age, leading to an increased risk of chronic disease, falling, functional decline and loss of independence.^4,5^ Traditional structured lifestyle interventions that utilise group or individual education, telephone counselling and behavioural change techniques can be effective at increasing physical activity levels in older adults.^6–8^ Unfortunately, physical activity levels tend to decline once participation in these types of interventions ceases, resulting in the health benefits being lost.^9^ Telephone counselling is an established method of providing ongoing support and can assist in the maintenance of physical activity levels for up to 24 months. Although effective, telephone counselling is both labour and resource intensive,^10^ and has not been widely adopted in clinical practice.^11^

An effective and, potentially, more cost-effective^12^ method of delivering ongoing feedback and support is achieved by utilising a wearable activity tracker. Wearable activity trackers have been shown to increase short-term physical activity participation across a range of populations, including healthy adults, chronic disease populations and older adults.^13–16^ Activity trackers, and their associated mobile applications (apps), are unique in that they provide users with real-time feedback about their physical activity participation, including a range of behaviour change techniques (BCTs), and do not require trained health professionals to deliver it. However, to receive the feedback, users must be able to operate the trackers and apps, and the feedback provided is generally generic in nature, which may influence the user’s perceptions and habitual use of the technology.^17^ By combining feedback delivered by a health professional with the feedback provided by an activity tracker, participants may feel more engaged.^18^

Research has shown that activity trackers are found to be well-accepted by various populations ranging from adolescents^19^ to older adults,^20,21^ as well as chronic disease and cancer populations.^22–24^ But to fully understand whether an activity tracker can support people, particularly older adults, to be habitually physically active, and to help us best utilise these devices, we need to fully understand the user’s experiences of activity trackers over long-term use.

Many activity tracker feasibility and acceptability studies include short follow-up periods of 12 weeks or less.^22,24,25^ Longer-term activity tracker use has been explored in existing users,^26^ but this does not provide insight regarding the initial adoption of an activity tracker. As the main goal of introducing an activity tracker is to encourage long-term behaviour change, it is difficult to gauge the true acceptability and perceived useability of an activity tracker when it is only worn for a short period of time. A longer-term follow-up allows for investigation into the way an activity tracker can provide ongoing support and promote positive habits for the long-term adoption of physical activity. Additionally, the role that a health professional can play in providing additional feedback based on the data recorded by the activity tracker has not been investigated.

Using thematic analysis^27^ of focus group data in addition to wear time of the activity tracker, the current study aims to explore older adults’ experiences of a wearable activity tracker combined with ongoing health professional support and feedback via text messages to assist them to maintain physical activity levels following a structured lifestyle intervention. The specific research questions were; (a) did the participants in the randomised controlled trial (RCT) like using the activity tracker; and, (b) did the activity tracker combined with health professional feedback, assist participants to achieve their physical activity goals?

## Methods

### Participants and design

**Figure 1.**
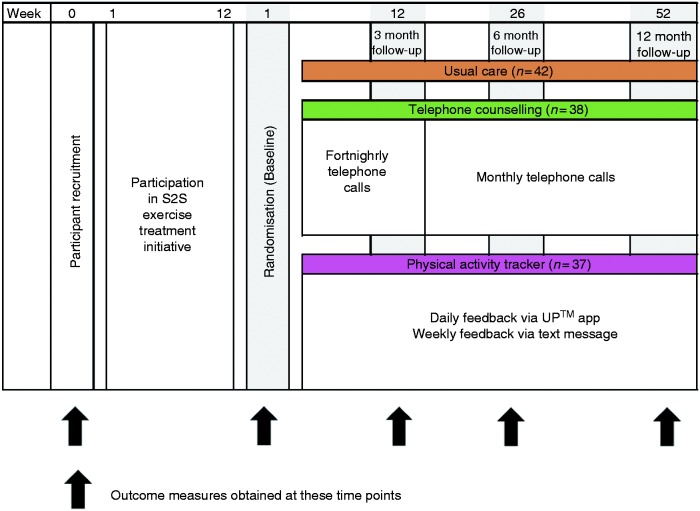
Study design indicating time points for participant recruitment, randomisation, data collection. All intervention groups included usual care. Note that outcome measures were also obtained at the commencement of Strength2Strength (S2S) but not used in the analysis of the randomised controlled trial (RCT).

Participants were originally recruited to take part in a 12-month RCT examining the effects of different types of feedback on physical activity maintenance following a 12-week structured exercise and lifestyle programme. The Strength2Strength (S2S) programme was an accredited exercise physiologist (AEP)-led individually tailored exercise programme aimed at older adults and those with chronic conditions. The protocol for the larger study has been described elsewhere.^12^ Briefly, the study compared the use of telephone counselling (TC), a consumer-based wearable activity tracker (AT) and usual care (UC) on maintenance of physical activity levels in older adults. Constructs from social cognitive theory (SCT)^28^ were included in both the TC and activity tracker intervention groups and included the provision of feedback, self-monitoring and goal setting. To be eligible for the study, participants had to have been referred to and completed the 12-week S2S programme. Participants were recruited during the initial S2S assessment and then randomised to one of the three intervention groups during the final S2S assessment (Figure 1). For the purpose of evaluating the interventions, data was collected at baseline (end of S2S) and at three, six and 12-months post-intervention commencement. Only the wearable activity tracker group is examined in this study. All intervention groups (including UC) received a home exercise programme and optional referral to an appropriate community-based exercise programme at the completion of S2S. The TC group received fortnightly phone calls for the first 3 months of the intervention from an AEP and then monthly for the remainder of the intervention. Those in the AT group received a Jawbone UP24 and a mobile device (Telstra Buzz, ZTE, China) with the Jawbone UP app downloaded. Participants also received weekly text message feedback relating to their daily step counts from an AEP. The study was approved by the Tasmanian Health and Medical Human Research Ethics Committee (H0014713). All participants provided written informed consent for the 12-month intervention, with additional consent required for participants attending the focus groups. All individuals who completed the 12-month activity tracker intervention were invited to take part in the focus groups.

### Jawbone UP24 Activity Tracker

**Figure 2.**
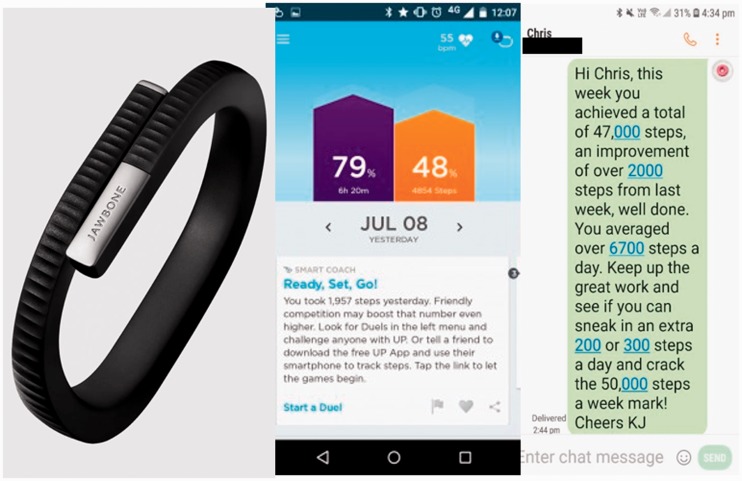
The Jawbone UP24 activity tracker, an example of the Jawbone UP application (app) user interface showing progress towards daily step count, and an example text message sent to activity tracker) activity tracker intervention participants.

The Jawbone UP24 activity tracker and mobile device (with data plan) was provided to participants randomised to the activity tracker group. The Jawbone UP app was downloaded to the mobile device, allowing participants to synchronise and view their daily step count (Figure 2). Participants were instructed to wear the activity tracker on their non-dominant wrist. A separate Jawbone account was created for each participant, allowing the AEP to remotely access participants step data and provide feedback based on daily step counts. The activity tracker was programmed to give a vibrotactile alert when the daily step goal was achieved. The daily step goal was determined based on the functional capacity and activity level of the participant at time of randomisation. An idle alert was also programmed to provide participants with a vibrotactile alert if they had not recorded any steps during a 30-minute period. At the time of randomisation, an individual information session was provided to teach the participant how to use the activity tracker, how to synchronise it with the mobile device and view their daily steps and how to charge the device. Written instructions were also provided. Participants were asked to synchronise the activity tracker with the UP app at the end of each day and to charge the activity tracker overnight. As there is no display on the Jawbone UP24 activity tracker, participants were required to use the UP app to view their progress towards their daily step goal. The UP app provides additional features including insights into length of active or inactive time, weekly activity trends and healthy lifestyle hints and tips. Participants were free to engage with these features as much as they wished. In addition to the feedback provided by the activity tracker and app, participants received weekly text messages from an AEP. Text messages contained feedback relating to average daily step counts, comparison to their daily step goal and the previous week (Figure 2). Participants meeting their daily step goal were encouraged to maintain their activity levels, while those who were not meeting their daily step goal were encouraged to increase their steps by an achievable amount each day. Telephone and in-person support were provided as required. Table 1 shows the feedback provided to participants throughout the intervention and the related behavioural change constructs. Participants did not receive any financial incentives for participation in the study, they were, however, able to keep the activity tracker at the end of the intervention.

**Table 1.** Timing and type of feedback delivered throughout the activity tracker intervention. Text in *italics* indicate a behaviour change construct.

	Feedback provided to participant	Researcher Tasks
Week 0 (Randomisation)	• Participant provided with activity tracker• Initial instructions on how to use activity tracker ad mobile device provided• Initial step *goal* established	• Provision of activity tracker and instructions• Determine appropriate step *goal*
Weeks 1–52	**Daily**• *Self-monitoring* via UP app• *Feedback* from app**Weekly**• *Feedback* from health professional via text• Updated *goal setting*	• Retrieve and consolidate daily step counts from previous week• Provide *feedback* and encouragement via text message• Update daily step *goal* as required

### Data collection

Demographic and health data were obtained at the start of the RCT and included participant age, body mass index (BMI), diagnosed chronic conditions and levels of activity. The primary outcome measure of the RCT was daily steps as measured by an ActivPAL accelerometer. Acceptability data relating to participants wear time of the Jawbone and any ongoing support provided to the participants was also examined. Non-wear days were defined as days on which no step data was recorded.^29^ The remaining days were considered to be ‘wear days’ and were used to calculate the percentage of days in which the activity tracker was worn throughout the 12-month intervention.

Participants were asked to complete a questionnaire prior to the focus group to obtain information relating to participants use of technology, any support they receive to use technology and their level of education (Supplementary Material Figure 1). Data from the questionnaire was summed and percentages calculated for each category.

A total of four focus groups were conducted and digitally recorded. The number of included participants ranged from 3–7 participants per group. Focus groups (mean duration 46.5 min, standard deviation (SD) 8.9 min) were transcribed verbatim, producing 74 pages (Times New Roman, size 12) of raw transcription data for further analysis. All focus groups were run by a researcher (JO) with experience in facilitating focus groups, who had not previously had any direct contact with the research participants, to ensure participants felt comfortable expressing their opinions. The primary researcher (KB) provided the focus group facilitator with semi-structured questions with prompts utilised as required. Table 2 contains the guiding questions utilised by the focus group facilitator.

**Table 2.** Guiding questions utilised during focus groups.

1.	Tell me about your previous experience with technology.
2.	Think back on your experience with the activity tracker – can you describe the experience? Was the activity tracker easy to use? If not, what did you find difficult about using the tracker? What did you like/dislike about the activity tracker?
3.	I’d like to hear more about it you think that activity tracker helped increase your activity levels. What features helped/didn’t help? If not a specific feature of the tracker, what was it that motivated you to be more active? What other features do you think would help to motivate you?
4.	Do you think receiving regular feedback helped keep you on track? Why did/didn’t this work for you?
5.	What did others think of you using an activity tracker?
6.	Did you continue to use an activity tracker after the study had finished? Why/why not?
7.	Do you have any recommendations for improvement?
8.	Any other comments?

### Data analysis

Transcripts were coded and thematically analysed in NVivo using a qualitative descriptive approach.^30^ Coding was completed in two stages. During the initial stage, two researchers (KB and JO) independently coded the transcripts inductively to ensure that themes were organically identified without researcher bias. Stage 2 involved two researchers (KB and JO) generating a preliminary list of themes which were then discussed and refined to ensure that all themes captured those previously coded. The two authors independently coded the transcripts to ensure all relevant data was captured. Key quotes were then selected to highlight the identified themes. A list of nodes and themes identified during Stages 1 and 2 is available in Supplementary Material Figure 2.

## Results

### Demographics

A total of 20 of the 28 participants who completed the activity tracker intervention consented and took part in the focus groups (females *n* = 12, males *n* = 8, age 73.6 ±5.5 years, BMI 31.4 ± 6.3 kg/m^2^). Participants were completing an average of 7098 ± 3384 steps per day and had a range of diagnosed chronic conditions including cardiovascular disease, diabetes, osteoarthritis and pulmonary conditions. All participants had at least one diagnosed chronic condition, with 65% (13 out of 20) participants having two or more diagnosed chronic conditions. Only one participant reported not using technology. The remaining participants reported using a mix of devices in their day-to-day lives, with mobile phone use more prevalent than smartphone use (50% compared to 30%). For participants who reported receiving support, the majority (55%) relied on friends or family with only one participant reporting that they sought professional help.

Participant demographics are outlined in Table 3.

**Table 3.** Participant baseline demographics.

	Mean ± SD	Range
Age (years)	73.6 ± 5.5	63.5–82.8
BMI (kg/m^2^)	31.4 ± 6.3	21.2–43.7
Daily steps	7098 ± 3384	1976–14,452
Activity tracker wear		
– Days	316 ± 49	201–364
– Percentage of total time	86%	55–99%
Diagnosed chronic conditions - *n* (%)		
– Cardiovascular disease	13 (65%)	
– Diabetes	4 (20%)	
– Osteoarthritis/osteoporosis	16 (80%)	
– Pulmonary	2 (10%)	
Devices regularly used^a^ - *n* (%)		
– Mobile phone	10 (50%)	
– Smartphone	6 (30%)	
– Tablet	9 (45%)	
– e-Reader	4 (20%)	
– Laptop/computer	16 (80%)	
– None	1 (5%)	
Support - *n* (%)		
– Family/friends	11 (55%)	
– Professionals	1 (5%)	
Highest level of education - *n* (%)		
– Less than year 12	10 (50%)	
– Year 12 or equivalent	4 (20%)	
– Vocational	2 (10%)	
– Associate diploma	2 (10%)	
– Undergraduate degree	1 (5%)	
– Postgraduate degree	1 (5%)	

BMI: body mass index; SD: standard deviation.

a Regular use refers to devices used at least once a week on an ongoing basis.

### Activity tracker use and support

Data from the activity tracker reflected that the Jawbone was worn by participants for an average of 86% of the available days (316 days out of 365). The number of non-wear days ranged from 1–164 days. The primary reasons for not wearing the band included forgetting to put the band on (all participants on at least one occasion), away on holidays (*n* = 5), damaged/broken band (*n* = 9), poor health/hospitalisation (*n* = 6) and technical issues requiring researcher support (*n* = 16). Telephone and in-person support were provided to participants as required over the 12-month intervention. In-person support was provided on a total of 27 occasions for the following reasons; Jawbone would not synchronise with mobile device (*n* = 15), Jawbone was damaged or stopped working (*n* = 10) or the mobile device was damaged or stopped working (*n* = 2). A total of 10 activity trackers were replaced over the 12-month intervention period. In addition to the provided in-person support, telephone support was provided on 23 occasions. The primary reasons for telephone support was that the mobile device had accidentally been switched onto ‘airplane’ mode or Bluetooth had been switched off.

### Acceptability

The high rate of Jawbone use suggests that the Jawbone activity tracker was well-accepted by older adults (Jawbone was worn on an average of 86% (316 out of 365) of possible days). The majority (80%, *n* = 16) of participants used words such as ‘encouraged', ‘good' and ‘motivating' to describe the Jawbone activity tracker, indicating an overall positive experience for most participants. ‘It was good, yeah it encouraged you to do it’ (Female, 75 years). ‘I thought it was good, was fantastic to have it. Since I’ve got the Jawbone it’s made me get up and do things’ (Male, 63 years).

### Thematic analysis

Four themes were identified in the thematic analysis of the focus groups and included: increased sense of awareness of activity levels is related to motivation; the level of engagement with the activity tracker influences the user experience; the role of feedback from a health professional in providing ongoing support; and the role of habits in supporting long term behaviour change.

## Increased sense of awareness of activity levels is related to motivation

Most participants (85%, *n* = 17) reported that using the activity tracker, made them more aware of their physical activity levels and sedentary behaviours. For most of the participants, this increase in awareness led to increased motivation.

I think it’s a really good way of knowing what you’re doing and if you’re doing enough, because if you’re aware of it and you’re not then you can up it. (Female, 66 years)

I found that if I didn’t have my steps up, 10,000 a day, when anybody rang me I never sat down and spoke, I walked around the house. I just kept walking and I got the steps up. (Female, 74 years)

No, it’s just as I said, as a reminder all the time, yes. You’re aware of it because it’s there, but it encourages you to do a little bit more than you probably would have before. (Male, 82 years)

However, there was one participant who felt that the awareness of their activity levels had a negative impact as it highlighted their reduced capacity.

It started off my health was reasonable, it wasn’t perfect, but it was reasonable, and I could get out and walk of a day. And then my health deteriorated, and I can’t walk unless I’ve got somebody with me. And also, my walking has reduced to very limited, my steps and everything like that. So I was getting more depressed about that happening to me because I was under that pressure to try and do what was asked of me. So that sort of made me realise more that my ability to walk was reducing. (Female, 73 years)

Despite the negative experience reported by this participant, the same participant made the following comment;

But at the same time there was – even though it was negative to me – it was a motivation too. Because otherwise I wouldn’t be cranky that I hadn’t enough steps up. Just pushed myself a little bit harder. (Female, 73 years)

## The level of engagement with the activity tracker influences the user experience

The way in which participants engaged with the activity tracker differed between participants and was influences by the following identified subthemes; (a) previous experience with technology (b) accuracy of the activity tracker and (c) and the design of the activity tracker.

### Previous experience with technology

Some participants felt more comfortable than others in exploring the device and associated Jawbone UP app.

Yeah, there was all this other stuff that you could find out, that was … but the actual activity thing, and what it decided was your sleeping time I found quite fascinating. (Female, 68 years)

There are features of this thing, active time, longest active time, longest idle time. Longest active time at the moment is 10 minutes, longest idle is 1 hour, 5 minutes. (Male, 71 years)

Others did not feel comfortable exploring the Jawbone UP app and therefore did not engage with the additional information that was available to them.

Unless it was there I didn’t touch mine, ‘cause I know if I touch anything electrical, you’ve gotta call someone to come and fix it. (Female, 72 years)

Perfectly all right as far as the band was concerned, but I was always afraid of pressing the wrong button in the app, or whatever, and occasionally it would go funny. (Male, 80 years)

### Accuracy of the activity tracker

Some participants questioned the accuracy of the device, reporting that not all the activities they did throughout their day seems to ‘count’ towards their steps. This influenced their experience with the activity tracker as it resulted in some frustration that their efforts were not being recognised.

I would question it, but I must have done much more than that much, ‘cause I was on my feet for 6 hours. (Male, 75 years)

And I would go do my exercise class and they would make us work so hard, and I would read the thing that night and it’d be such a limited number of steps for the amount of effort that I’d put into it. (Female, 73 years)

And I did lose interest when it wasn’t working [band wouldn’t always record steps on treadmill]. (Female, 74 years)

### Design of the activity tracker

In addition to the accuracy of the device, some participants reported not liking the design of the activity tracker which again caused some frustration.

I found it wasn’t quite robust enough, it would catch on stuff. I had a couple of panics where it was gone (Male, 75 years)

Yes, it would catch on things, and towards the end it was getting quite lose and it was a pain ‘cause I like my big watch, and it used to get caught underneath it. Mine pulled off in the garden, a few times. I hauled it out of the compost bin at least once! (Female, 68 years)

## The role of feedback from a health professional in providing ongoing support

As previously mentioned, the focus group participants acknowledged an increased level of awareness of their physical activity behaviours just from wearing the activity tracker itself. However, they also made repeated references to the support received from the AEP who set up the activity trackers and sent the weekly text messages. In addition to the direct support provided by the AEP, participants referred to feeling as though they were being ‘watched’, which they felt helped to keep them on track. Some participants also appeared to feel as though they were letting the AEP down if they did not keep up their activity levels. Having the additional support provided by the AEP appeared to enhance the overall user experience.

It was knowing you were connected up to somebody else. As I said, thinking of someone watching over me, not mentioning any names. No just thinking how well she’d done, then to let It go, it’s not fair to the programme and it’s not fair to me either. (Female, 73 years)

I didn’t want to feel as though I was going backwards. I had to go back to Katie-Jane (AEP) for another appraisal, so that’s probably why I kept it up. I didn’t want to be going backwards. (Female, 70 years)

I felt it was doing me a whole heap of good, like somebody was always looking over my shoulder and keeping me (on track). (Male, 71 years)

Yeah it was great [the text messages], I looked forward to every Monday to see what her remarks were. See whether I’d done good the week before, or you’d done bad. You know sometimes if I was a bit down, I’d ring her up and tell her I hadn’t been well the last couple of days. She’s not pushing anyone to do it, she always explained to you, it’s up to you how you do it. (Male, 63 years)

It encouraged me. I don’t know that it kept me on track, but I was really chuffed when I got a good, oh well, a very good [report]. (Female, 79 years)

I thought it was great [the text messages]. I mean, yeah okay I knew when I didn’t do my steps, but it sort of prompted me – well I’ll do better tomorrow. (Female, 69 years)

## The role of habits in supporting long-term behaviour change

Throughout the 12-month intervention, participants established a number of habits that helped them to achieve their daily step count. Participants reported that towards the end of follow-up period, they felt that they had established some effective habits in relation to their activity levels and no longer felt they ‘needed’ an activity tracker.

I’d think to myself, Oh God, can’t I have a day off without getting in my face all the time, but really I know myself, if they hadn’t I’d think oh well, I can just do what I want and it wasn’t any good for me, because now, that I haven’t got it [the activity tracker] I still do it. Also, if I don’t, sometimes I get a pain in my lower back and I know then it’s because I’ve been sitting, when it’s been pouring with rain and you can’t get out. But now, because I know how far I’ve gotta go every day, I don’t really need a tracker anymore. (Female, 72 years)

It’s a good way of knowing what you’re doing. But I wouldn’t wear it like 12 months or two years. I might wear it for a few months to see how I’m going, and then I think oh, I’m not doing so good, I’d better walk more or whatever. But I know trackers, people love them, they have them on whenever they walk, when they ride, when the swim, whatever they’re doing, but I would like to be able to, well not force myself, but make myself do these activities not because I’m wearing a tracker. Just because it’s the right thing to do, it’s good more me, it’s health for me. I don’t want good job, good job, you know? I know it’s good for me, but to do it without that push now, because I’ve had the push for 12 months, now I’d just like to do it under my own steam because it’s good for me. (Female, 66 years)

One participant reported that the activity tracker helped her to establish some habits that allowed her to increase her daily physical activity levels.

And if I didn’t get my steps up, when I was in the bathroom at night I’d do a few steps or a few bits of exercise. And when I watched tele at night, every time there was an ad on I’d run up to the bathroom, I’d clean my teeth that time, go back, watch the next part of the show. Next ad, I’d go up and put my nightie on. Next time I kept going back to the bathroom in the ads, so I kept walking. (Female, 74 years)

## Discussion

To our knowledge, this is the first study to look at the experiences of the Jawbone UP24 activity tracker in older adults with various chronic conditions during a 12-month RCT. Other studies have examined the use of activity trackers including the Jawbone UP24 in older adults, however wear time of device was less than a week.^22^ Other trackers have been evaluated in older adult populations over longer time periods, with experiences evaluated via questionnaire^20^ or telephone interviews.^31^

### Acceptability

Similarly to previous research, the use of an activity tracker was well-accepted by the study participants.^22,23,32^ Overall, participants reported that the activity tracker was easy to use and was helpful in assisting them to increase or maintain their activity levels. This is supported by the results of the RCT, which demonstrate an initial increase in daily step count between baseline and three-months and maintenance of daily steps between baseline and 12-months (data not yet published). While the current study required participants to wear the activity tracker for a 12-month period, the median number of days the activity tracker was worn is comparable with previous research in older adult populations (88% compared to between 93% and 95%).^23,33^ Despite having a wear time of between 4–12 weeks, the comparable wear time found in the current study infers that the use of wearable activity trackers can be sustained over prolonged periods of time. Interestingly this differs from younger populations, in which wear time can be as little as 15%.^17^

### Thematic analysis

One of the common themes identified in the current literature^22–24^ is an increase in self-awareness resulting in increased motivation to improve or maintain physical activity behaviours. While this theme was also identified in the current study, we found that the increased awareness did not necessarily translate to increased motivation. This was due to one participant reporting that she found the increased awareness of her reduced activity levels due to declining health depressing and discouraging. As a result, the theme included in the current study links awareness to motivation, however no direction (increase or decrease) is applied to the motivation. While this finding has not previously been reported in older adult populations, negative feelings associated with the pressure of achieving a daily step target have been reported in adolescent populations.^19^ It is worth noting that the tailored feedback provided in this instance did not allay the ongoing difficulties of the participant. As such, exploring the potential ‘demotivating’ effects of an activity tracker and what additional support can be provided to reduce the likelihood of this occurring would be beneficial.

The level of engagement with the activity tracker has also previously been identified in the literature.^22,24,31^ It is well established that if an individual believes that technology will improve their life or be easy to use, they will be more likely to adopt the technology.^34^ While most participants indicated that they found the activity tracker easy enough to use, the level of engagement differed between participants depending on several factors. The extent to which the individual felt comfortable exploring the activity tracker features varied depending on their previous experience with technology, a finding which has been previously reported.^22,24,35^

Both the perceived accuracy and design concerns have previously been highlighted in the literature and are not unique to the Jawbone UP24 activity tracker.^22–24,35^ While the Jawbone UP24 activity tracker has been validated in older adults,^36^ wrist worn activity trackers have been shown to under-estimate steps during lower-intensity activities.^37^ Irrespective of the demonstrated accuracy of an activity tracker, if the participant perceives that the activity tracker is not accurate, it can lead to feelings of frustration and loss of interest and motivation. Although it is not possible to design an activity tracker that suits all individuals, considerations should be given to the activity tracker design and demonstrated accuracy within specific populations when using in research or in clinical practice. Furthermore, it is vital to provide participants with instructions to suit the individuals level of technology literacy. Interestingly, despite there being a number of technical issues with the Jawbone UP24 activity tracker, this was not highlighted during the focus groups as it has been in previous research into older adults experiences.^24^ Participants had access to technical support from the AEP delivering the intervention, meaning that technical issues such as the band failing or not synchronising which may discourage participants from using the band^31^ were promptly addressed and therefore did not appear to disrupt the overall experience of the user.

The feedback provided to participants from the AEP appeared to have a significant effect on the way in which participants engaged with the activity tracker. By knowing that there was a ‘real’ person monitoring their activity levels, participants reported a feeling of being watched and were therefore potentially externally motivated to achieve their daily step goal. External motivation does not generally translate well to long-term behaviour change,^38^ so further research is required to determine if this type of health professional/participant relationship is feasible in the long-term. This theme has not previously been identified in the literature, most likely as the study designs were purely focused on evaluating the activity tracker itself and did not include the level of researcher interaction in the current study. However, previous research indicates that participants expressed interest in sharing their activity tracker data with healthcare professionals.^22,39^

The formation of habits can help to predict the adoption of physical activity behaviours,^40^ with the integration of daily habits assisting in the long-term usage of an activity tracker.^31^ It is therefore positive to see that participants were able to create habits that assisted them to achieve their daily step count. Some participants identified that they no longer felt they ‘needed’ an activity tracker, suggesting a level of intrinsic motivation. Intrinsic motivation is well recognised as one of the most important aspects of long-term behaviour change^41^ and has been highlighted in previous research as an important aspect for the long-term adoption of activity trackers.^35^

## Strengths and limitations

A key strength of the current study is that included participants had been wearing the activity tracker for a 12-month period, therefore reducing the impact of any novelty effect of wearing the activity tracker. Included participants had a range of chronic health conditions and had varying mobility limitations. Furthermore, there were no inclusion criteria relating to previous technology experience, making the findings more transferable to the general older adult population. The experiences of older adults utilising activity trackers have been widely explored,^42^ however the addition of ongoing feedback from a health professional in addition to the use of activity tracker has not been investigated. While incorporating the feedback from the activity tracker and the health professional is a limitation in the sense that it is not possible to separate the effects of each type of feedback, it is also a strength of the study. The experience of the participants appears to be enhanced through the inclusion of health professional feedback and may have contributed to the overall positive experience of participants. Additionally, using a researcher who had no previous contact with the participants to run the focus groups encouraged open and honest discussion about their experiences with the activity tracker.

There are however several limitations to the current study. The design of the Jawbone UP24 activity tracker means that participants were required to view the app in order to view their step progress. As such, the experience of the activity tracker itself and the app are intertwined. As most activity trackers are supported by an app, this is likely to occur across a range of different commercially available activity trackers. While the Jawbone UP24 is no longer available for purchase, the themes identified in the current study, including those relating to the accuracy and design of the Jawbone UP24 are not unique to this activity tracker, with similar issues reported in other activity tracker brands.^22^ Recommendations for research include the use of open-ended questions during focus groups, or potentially exploring the use of multiple methods than can better capture the integration of qualitative research with the setting of RCTs.^43^

## Conclusion

This is the first study to examine the long-term use of an activity tracker combined with health professional feedback in older adults with a range of chronic health conditions. Although the link between awareness and motivation has previously been identified, we found that in some instances, increased awareness may contribute to decreased motivation and may not have the positive effects previously reported. As such, we recommend that where possible, feedback provided to older adults via the activity tracker should be individualised and targeted to accommodate changes in an individual’s health status or functional capacity. A unique feature of the current study was the provision of feedback by a health professional, resulting in the identified theme relating to the role health professionals play in providing ongoing support. While this needs to be further explored to determine feasibility, it appears that combining technology with health professional support may improve the user experience and level of engagement of participants.

## Supplemental Material

sj-pdf-1-dhj-10.1177_2055207620921678 - Supplemental material for Older adults’ experiences of using a wearable activity tracker with health professional feedback over a 12-month randomised controlled trial Click here for additional data file. Supplemental material, sj-pdf-1-dhj-10.1177_2055207620921678 for Older adults’ experiences of using a wearable activity tracker with health professional feedback over a 12-month randomised controlled trial by Katie-Jane Brickwood, Andrew D Williams, Greig Watson and Jane O’Brien in Digital Health
