# Supplementary material for: Older adults’ experiences of using a wearable activity tracker with health professional feedback over a 12-month randomised controlled trial
Source: Digit Health. 2020 Apr 26;6:2055207620921678. doi: 10.1177/2055207620921678 (PMC7218318; doi:10.1177/2055207620921678)
Supplement: sj-pdf-1-dhj-10.1177_2055207620921678 - Supplemental material for Older adults’ experiences of using a wearable activity tracker with health professional feedback over a 12-month randomised controlled trial [file sj-pdf-1-dhj-10.1177_2055207620921678.pdf]

Strength2Strength Physical Activity Tracker Focus Group

Questionnaire

Please complete the following questions to the best of your knowledge. These questions will provide us with some background information which will help us to interpret and understand the information collected during the focus group.

Please circle the most appropriate answer(s).

1. ***Do you regularly use technology?***

   If yes, what do you use?

Mobile Phone Smartphone Tablet e-Reader Laptop Computer

2. ***If you do use technology, do any have any support to do so?***

   Family Friends Professionals Other (please specify) ___________

3. ***What is your current living situation?***

Live alone with spouse/partner with children/grandchildren with friend/s

Other (please specify) _______________________

4. ***What is your highest level of education?***

Less than year 12

Year 12 or equivalent

Vocational qualification

Associate diploma

Undergraduate diploma

Bachelor Degree

Postgraduate Degree

Master's degree

Doctorate

NAME:____

Supplementary Figure 2 – Node and Themes identified during NVivo coding

Nodes

Name	Files	References
Phase 1	0	0
Activity Tracker	0	0
Accuracy	4	19
Activity Tracker - Neg	3	9
Activity Tracker - Posi	3	12
Design of tracker	4	18
Ease of Use	2	3
Feedback from tracke	4	18
Idle Alert	2	15
Monitoring	3	15
Motivation Encourag	4	38
Not motivating	3	6
Other features of the	2	8
Remembering to wea	4	23
Technical Issues	4	9
Continued Use	4	14
Family & Friends	4	18
Health Benefits	1	4
Individual Goals	1	1
Previous Technology Exp	4	38
Pride	1	1
Recommendations	1	1
Support	3	7
Text messages	3	9
Phase 2	0	0
Awareness - Increased se	4	65
Engagement - The level	4	54
Feedback - The Importan	4	33
Habits - The role of habit	4	23
